# Offering Self-administered Oral HIV Testing as a Choice to Truck Drivers in Kenya: Predictors of Uptake and Need for Guidance While Self-testing

**DOI:** 10.1007/s10461-017-1783-9

**Published:** 2017-05-24

**Authors:** Elizabeth A. Kelvin, Gavin George, Eva Mwai, Eston N. Nyaga, Joanne E. Mantell, Matthew L. Romo, Jacob O. Odhiambo, Kaymarlin Govender

**Affiliations:** 1 Department of Epidemiology and Biostatistics and Institute for Implementation Scince in Population Health, CUNY Graduate School of Public Health and Health Policy, City University of New York, 55 West 125th Street, New York, NY 10027 USA; 2 Health Economics and HIV and AIDS Research Division, University of KwaZulu-Natal, Durban, South Africa; 3 North Star Alliance, Nairobi, Kenya; 4 Division of Gender, Sexuality and Health, Department of Psychiatry, HIV Center for Clinical and Behavioral Studies, New York State Psychiatric Institute & Columbia University Medical Center, New York, NY USA

**Keywords:** Diagnostic tests, HIV, HIV testing, Kenya

## Abstract

We assessed predictors of choosing self-administered oral HIV testing in the clinic with supervision versus the standard provider-administered blood test when offered the choice among 149 Kenyan truck drivers, described the types of guidance participants needed during self-testing and predictors of needing guidance. Overall, 56.38% of participants chose the self-test, 23.49% the provider-administered test, and 20.13% refused testing. In the adjusted regression models, each additional unit on the fatalism and self-efficacy scales was associated with 0.97 (p = 0.003) and 0.83 (p = 0.008) times lower odds of choosing the self-test, respectively. Overall, 52.38% of self-testers did so correctly without questions, 47.61% asked questions, and 13.10% required unsolicited correction from the provider. Each additional unit on the fatalism scale was associated with 1.07 times higher odds of asking for guidance when self-testing (p < 0.001). Self-administered oral HIV testing seems to be acceptable and feasible among Kenyan truck drivers, especially if given the opportunity to ask questions.

## Resumen

Evaluamos los factores predictores de elegir de la prueba oral de VIH autoadministrada en la clínica con supervisión versus la prueba estándar de sangre administrada por el proveedor cuando se les ofreció la elección entre 149 conductores de camiones en Kenia; también describimos la ayuda que los participantes necesitaron durante la autoadministración y predictores de necesitar ayuda. En total, el 56,38% de los participantes optó por la prueba autoadministrada, el 23,49% optó por la prueba administrada por el proveedor, y 20,13% rehusó hacer la prueba. En los modelos de regresión ajustados, cada unidad adicional en las escalas de fatalismo y autoeficacia se asoció con 0,97 (p = 0,003) y 0,83 (p = 0,008) veces menos Probabilidad de elegir la prueba autoadministrada, respectivamente. En total, el 52,38% de los participantes autoadministradores hizo la prueba correctamente sin tener preguntas, el 47,61% hizo preguntas y el 13,10% necesitaba corrección por el proveedor no solicitada por parte del participante. Cada unidad adicional en la escala de fatalismo se asoció con 1,07 veces mayores probabilidad de hacer preguntas entre los participantes autoadministradores (p < 0,001). La prueba oral de VIH autoadministrada parece ser aceptable y factible entre los conductores de camiones en Kenia, especialmente si se les da la oportunidad de hacer preguntas.

## Introduction

In 2014, the Joint United Nations Programme on HIV and AIDS (UNAIDS) launched its ambitious 90-90-90 HIV testing and treatment goals that by 2020, 90% of people living with HIV will know their HIV status, 90% of people diagnosed with HIV will receive sustained antiretroviral therapy, and 90% of people receiving antiretroviral therapy will have viral suppression [1]. A key motivation for these goals is the finding that treatment as prevention is effective in decreasing HIV incidence [2], and the World Health Organization (WHO) has recommended that all people living with HIV, regardless of CD4+ T cell count, should initiate treatment immediately for improved health and to decrease the probability of transmission to others [3]. However, HIV testing rates in many countries remain suboptimal, and in Kenya 2012–2013, only a little over half of those age 15–64 had tested in the past year and 53% of those HIV-infected were unaware of their HIV status [4]. New HIV testing strategies will likely be needed in order to reach the 90-90-90 goals

Truck drivers in Africa have been characterized as a key population to target for HIV prevention, testing and treatment services due to their high HIV risk and unmet need for services [5–7], and because they can be a conduit for the spread of HIV between female sex workers (FSWs) and other partners and across international borders due to work-related travel [6, 8]. Health clinics targeting truck drivers now appear along many major trucking routes [9–11], but the few available studies suggest that testing uptake in this population remains low. A 2003–2004 survey of 1896 long-distance truck drivers in South Africa found that only 38.2% had ever been tested for HIV [12]. In a 2009 study in a night clinic at a truck stop in northern Mozambique, only a quarter of participants accepted HIV testing when offered and, of those, 27% tested HIV+ [10]. A 2010 study among long distance truck drivers in Togo found 47.4% had ever tested for HIV [13]. In 2012, the North Star Alliance, an organization that runs 35 roadside wellness clinics providing services to truck drivers on major transit routes in Africa, reported only about 21% of 219,681 client-visits included HIV testing despite the fact that testing is offered at every visit [14]. Trucking Wellness, which runs 22 roadside clinics for truck drivers in South Africa, reported that only about 10% of the >90,000 clients seen in 2012 were tested for HIV, of which 7% were found to be HIV+ [15]. Thus, despite the convenience of roadside health facilities, demand for HIV testing remains low, suggesting that barriers persist.

On July 3, 2012, the United States (US) Food and Drug Administration (FDA) approved a rapid self-administered oral HIV test for at-home use (OraQuick In-Home HIV Test) [16]. This test can be used in or outside of a clinic setting, and has the potential to reduce a number of barriers to HIV testing faced by truck drivers, therefore potentially providing a more acceptable option for people in mobile professions who do not use existing HIV testing and counseling services. The ability to self-test at home or in private may allay concerns about the stigma of being seen at the clinic and possible breach of confidentiality, barriers often cited [17–20]. Self-administered testing in a clinic setting may also reduce the burden on healthcare staff, since staff is no longer performing the test, and thus shorten clinic waiting time; for those using the test outside of a clinic setting, there may be the added benefit of reduced travel and wait time and cost, although travel may be required to obtain the test kit. Furthermore, if the HIV test can be used outside of the clinic, truck drivers could pick up a test kit at a roadside clinic and test at home, which may be a less stressful environment [19, 21] and where they have access to their network of family and friends should they choose to test with a partner and/or discuss their testing experience and results with someone they trust.

Numerous studies have found self-administered HIV testing to be acceptable in African populations [22–29]. If this test will appeal to those at highest risk or those not testing under current testing programs, it has a great potential to increase testing rates. On the other hand, making such a test available might result in migration of those already testing to this new testing modality which might be less accurate than provider-administered testing when administered by someone with no training [30]. While there has been concern about migration from one HIV prevention method (condoms) to another (microbicides, medical male circumcision), there is limited evidence that such migration has or will occur [31]. Whether migration will be an issue for different testing modalities is unknown. Error rates in self-testing among African populations may also be a concern. In one study among South African healthcare workers, three of nine participants who self-tested HIV-positive incorrectly interpreted their tests as negative, resulting in a user sensitivity of only 66.7%, but 100% specificity. However, the authors argued that this could be improved with better instructions [27]. In another study among participants recruited from healthcare and workplace facilities in Kenya, the sensitivity of self-administration of the test was 89.5% and the specificity 99.4% when compared with a provider-administered rapid blood (finger-prick) test as the “gold standard” [23, 24]. A third study among a representative sample of suburban residents of Malawi found a sensitivity of 97.9% and specificity of 100% when compared with a provider-administered blood-based (finger-prick) test as the standard [26]. Ensuring support for confirmatory testing and linkage to HIV care for those who self-test HIV+ is another concern [30].

Whether truck drivers with certain demographic characteristics, or those at highest risk and/or not currently testing would prefer self-testing to provider-administered testing and whether they are able to administer the test and interpret the results correctly is unknown. In addition, it could be that truck drivers with certain psychosocial characteristics differ in the benefit they see to HIV testing in general or self-testing specifically, or in whether the offer of self-testing works as a cue to action (HIV test acceptance), two factors suggested by the Health Belief Model as impacting whether or not someone seeks screening [32]. Fatalism [33], anticipated HIV stigma [34] and low self-efficacy [35] have all been associated with decreased HIV testing in various populations, while low gender-equitable norms [36, 37] and sensation-seeking [38, 39] have been associated with increased HIV risk behavior in other groups.

Therefore, this paper explores potential predictors (demographic characteristics, HIV-related behavior, and scores on various psychosocial scales) of choosing a self-administered oral rapid HIV testing in the clinic with provider supervision versus the standard provider-administered blood-based (finger prick) rapid test among a sample of Kenyan truck drivers. We also describe whether the truck drivers were able to administer the test and interpret the results by themselves and the steps in the self-testing process where they sought or required guidance from the provider in order to administer the test correctly. In addition, we look at predictors of needing guidance when self-administering the oral HIV test.

## Methods

### Study Participants

We explored these secondary research questions using data from a randomized controlled trial evaluating if offering choices in HIV testing (provider-administered blood-based (finger prick) rapid HIV test or oral self-administered rapid HIV testing in the clinic with supervision or, only for those who refused both in-clinic testing options, a test kit for home use) versus the standard care (only offering the provider-administered blood test) would increase HIV testing uptake among truck drivers in Kenya. Participants were recruited from two North Star Alliance roadside wellness clinics located in Nakuru county, which has among the highest HIV prevalence in the country [40, 41]. The clinics provide primary healthcare services to key populations in Africa, such as truck drivers and sex workers, including HIV screening and treatment [9, 42]. Any male truck driver or trucking assistant (both referred to as truck drivers here) who visited the two clinics from October 2015 to December 2015 for services other than HIV treatment were informed by the receptionist that a research study was being conducted and were referred to one of the fieldworkers if interested for information and to be screened for eligibility. Eligibility criteria were: (1) at least 18 years old, (2) male (based on observation), (3) employed as a truck driver, (4) primary residence in Kenya, (5) able to speak English or Kiswahili, (6) self-reported HIV-negative or unknown HIV status (7) able to sign the consent form, and (8) willing to receive payment for participation fees via MPesa (a cell phone-based money transfer system widely used in Kenya). The study was described to potential participants as being about HIV testing experiences and preferences and they were told that HIV testing would be offered, as it would be at any North Star Alliance clinic visit, but their decision about testing would not impact healthcare services or study eligibility. Participants were not informed about the specific research question or the fact that they would be randomized to different HIV testing options in order to avoid bias.

Truck drivers who met eligibility criteria and consented to participate had the baseline questionnaire administered to them by the fieldworker, after which they were offered HIV testing, with the test offered depending on the study arm to which they were randomized. Those in the testing choice arm were given a brief demonstration of the self-testing kit before being asked to make their choice. Those who selected the self-administered oral rapid HIV test in the clinic had an HIV testing counselor (HTC) sit in the room while the participant self-administered the test. The participant was told he could ask questions and, if he did something incorrectly, the provider intervened with unsolicited instructions to ensure correct use of the test. The participant was also told that he could either view the test results with the HTC or alone in order to keep his test results completely private if he chose. Those who viewed the test results alone were encouraged to disclose the test results to the counsellor (who was, in most cases, the same person who supervised the testing) during posttest counseling to better tailor the counseling and referrals given, but if they chose not to disclose they were given posttest counseling and referrals for both an HIV+ and HIV− test result. Those who refused both in-clinic testing options were offered a test kit for home use with phone-based posttest counseling.

The study procedures were approved by the City University of New York Institutional Review Board, the Kenya Medical Research Institute Ethics Committee, and the University of KwaZulu-Natal Biomedical Research Ethics Committee.

The baseline interview included questions on demographics, HIV testing history and risk behavior. In addition, the questionnaire included a number of psychosocial scales on anticipated HIV stigma, fatalism, gender-equity, general self-efficacy and sensation-seeking (described below). All interviews were conducted in Kiswahili, English, or both, depending on the participant’s preference. The English version of the questionnaire was translated into Kiswahili and back-translated from Kiswahili into English to ensure accuracy of the translation. The HTC who supervised those who chose to self-test completed a form noting the steps in the process where the participant asked questions or where they had to intervene to ensure the test was administered correctly. Specifically, at each step in the testing process the HTC noted if the participant (1) did the step correctly without questions or correction, (2) did the step correctly but asked questions, (3) did the step correctly but only after unsolicited instruction from the counselor, or (4) did not do the step correctly despite instruction. The HTC also noted whether the participant asked to view the test result alone or with the HTC and, if alone, whether the participant disclosed the test results during posttest counseling.

## Measures

### Demographic Characteristics

We looked at a number of demographic characteristics. All participants were male and of African race, and therefore these variables were not included in the analysis. Age was collected and examined in years. Marital status was examined as an indicator for married (legal or common-law) versus not. Education was examined as an indicator for having completed at least high school (versus less than high school completion). Income from truck driver job was originally collected by asking “about how much money do you earn on an average month driving a truck?” and those who were unable or unwilling to specify their income were then asked, “could you tell me if your income is less than 8000 Kenyan Shillings (KES), 8000–16,000, 16,001–24,000, 24,001–50,000, or >50,001 KES?” The variable was dichotomized at about the first quartile into mid-high income (24,000–55,000 KES, as 55,000 KES was the highest income reported, which is about $235–$550 US dollars) versus less.

### HIV-Related Behaviors

We looked at a number of HIV-related behaviors. We included an indicator for having regular partners along the participant’s usual trucking route (road wives). We explained what we meant by saying “Men who travel for work often develop long-term romantic relationships with women or men in the towns through which they travel. By romantic relations I mean romantic attachments that may include vaginal or anal sex or other sexual behaviors such as oral sex or mutual masturbation. Do you have any regular partners, men or women with whom you have romantic relations, other than a wife or main partner at home, who you see on a regular basis, such as when traveling through their town on your regular route?” We also examined whether the participant had paid for sex in the past 6 months, and whether the participant had always used condoms when having sex during the past 6 months. Only five participants reported not having been sexually active in the past 6 months and those were coded into the no risk group (i.e. no partners along the trucking route, had not paid for sex in the past 6 months, and had always used condoms when having sex during the past 6 months). Alcohol consumption was examined based on response to the question “In the past year, how often have you had a drink containing alcohol?” Responses were dichotomized into an indicator for any alcohol consumed (versus none), which split the participants about in half. We also asked about HIV testing history and years since last tested, with those who had never tested having their age assigned to this variable (i.e. the years since last test was coded as their age since they had never tested).

### Scales

We looked at five different psychosocial measures.

1. Anticipated HIV stigma: We used a nine-item anticipated HIV stigma scale that was adapted from the UNAIDS general population survey and the Department of Health Services AIDS module, and previously used in Botswana [43]. The scale presents statements about possible stigma-related scenarios if the participant was to test positive for HIV and others found out about his status (e.g., Do you think you would be treated badly by health workers?). Each item elicited a yes/no response, and the number of yes responses were summed for a possible score range of 0–9, with higher scores indicating more anticipated stigma. We allowed one missing response on this scale (three participants were missing one item) in calculating the summary score. The Cronbach’s alpha for the scale in this study was 0.80, indicating good internal consistency.
2. General self-efficacy: We used a ten-item general self-efficacy scale [44], with previous multicultural validation in Europe and Asia [45], which presented statements related to belief in one’s ability to cope with a broad range of stressful or challenging demands (e.g., I can always manage to solve difficult problems if I try hard enough). Response options were on a four-point Likert scale from ‘not at all true’ to ‘exactly true.’ Responses were summed for a possible score range of 10–40, with higher scores indicating greater self-efficacy. We allowed for one missing item in calculating the summary score (one person was missing one item). Cronbach’s alpha for the scale in this study was 0.90, indicating good to excellent internal consistency.
3. Fatalism: We used a 20-item fatalism scale [46] that elicited agreement to a series of fatalistic statements mostly related to health (e.g., If someone is meant to get a serious disease, it doesn’t matter what kinds of food they eat, they will get that disease anyway). Response options were in a five-point Likert scale ranging from ‘strongly disagree’ to ‘strongly agree.’ Responses were summed for a possible score range of 20–100, with higher scores indicating greater fatalistic views. Six participants were missing responses to one or two items on the scale and we allowed for two missing responses in calculating the summary score. The Cronbach’s alpha for the scale in this study was 0.94, indicating excellent internal consistency.
4. Gender-equity: We used a 24-item gender-equity scale (the Gender-Equitable Men scale [47]) that has been widely used in sub-Saharan Africa [48]. It consists of a series of statements related to relationships between men and women (e.g., There are times a woman deserves to be beaten) with response options in the form of a three-point Likert scale including ‘agree’, ‘partially agree’ and ‘do not agree.’ Responses were summed for a possible score range of 24–72, with higher scores indicating more gender-equitable attitudes. Eleven participants were missing responses to one or two items on the scale and we allowed up to two items to be missing in calculating the summary score. Cronbach’s alpha for the scale in this study was 0.88, indicating good to excellent internal consistency.
5. Sensation-seeking: We used a five-item sensation-seeking scale [49], previously adapted for use in South Africa [38, 39], with statements about self-perceived propensity for risk and pleasure-seeking (e.g., I would enjoy the feeling of jumping off a high cliff into a river below). Responses were elicited on a four-point Likert scale ranging from ‘not at all like me’ to ‘very much like me,’ with a possible score range of 5–20, with higher score indicating greater sensation-seeking. There were no missing items on the scale for any participants. Cronbach’s alpha for the scale in this study was 0.74 after dropping the first item to increase internal consistency.

### Statistical analysis

Because the aims of this paper are to identify predictors of the HIV test chosen when given a choice and describe the self-testing process, in this paper we only used data from those in the study who were offered HIV testing choices (i.e., those randomized to the choice arm who received the intervention to which they were randomized, n = 149; one person in the choice arm was excluded because he was not offered a choice of tests as per protocol based on his randomization assignment). Because we are only looking at acceptance of HIV testing in the clinic, those who refused HIV testing in the clinic but later took a test kit for home use were classified as in-clinic test refusers in this analysis.

We described the sample overall and by HIV test selected for in-clinic use (i.e., no test, provider-administered blood test, supervised self-administered oral test). To assess the significance of differences by HIV test selected we used a Pearson’s Chi square test for categorical variables (Fisher’s exact test for any variables with expected cell counts <5) and the Kruskal–Wallis test for numeric variables. We then used logistic regression to identify predictors of choosing the supervised self-administered oral test over the provider-administered blood test among those who tested, conducting crude models looking at each independent variable alone, a full multivariate model with all the predictors included and, out of concern about the number of variables examined and possible type 2 error, we also conducted backward stepwise regression using p < 0.2 as the cut-off for inclusion in the final model. We described the self-administration of the oral HIV test among those who chose that test, including the steps in the process where the participants needed guidance, either by asking questions or requiring unsolicited instruction from the provider in order to administer the test correctly. Finally, we used logistic regression to identify predictors of needing guidance when self-testing among those who self-tested, following the same procedure described above to look at associations in the crude, full multivariate, and final backward stepwise models. All analyses were conducted in SPSS version 23 (Chicago, IL) unless otherwise specified in the tables, with p-values <0.05 considered statistically significant and p-values between 0.05 and 0.15 considered borderline significant.

## Results

### Description of the Sample

A total of 149 study participants were offered HIV testing choices. Mean age was 37.07 years and 38.26% had completed high school. Three-fourths of the participant earned between 24,000 and 55,000 Kenyan Shillings per month from their truck driving job, on average (about $240–$550 US) and 82.99% were married. Almost half (48.30%) of participants had regular partners on their trucking route in addition to their wives or main partners at home, 52.86% had paid for sex in the past 6 months and only 15.26% reported they had always used condoms during sex in the past 6 months. Half of participants (51.68%) reported drinking alcohol during the past year. Only 10.07% reported never having been tested for HIV and the mean years since last HIV test was 4.74. Participants had a low anticipated HIV stigma score, with a mean of 0.80 and median of 0.00 on a scale of 0–9. Their mean score on the fatalism scale was on the lower-mid range at 46.62 on a scale of 20–100, as were their scores on the gender-equity and sensation-seeking scales (mean = 58.36 with a possible range of 24–72 and mean of 1.38 with a possible range of 4–16, respectively). Participants scored high on the general efficacy scale, with a mean of 36.64 out of a possible range of 10–40. (Table 1).

**Table 1:** Description of the participants overall and by test selected

Characteristics	Total, n (%)	Refused HIV testing, n (%)	Chose provider-administered blood test, n (%)	Chose self-administered oral test, n (%)	X^2^ statistic, unless otherwise specified	P-value
Total	149 (100)	30 (20.13)	35 (23.49)	84 (56.38)		NA
Demographics						
Age in years					0.086*	0.958*
Mean (SD)	37.07 (7.79)	36.63 (7.49)	36.63 (7.44)	37.40 (8.10)		
Median (range)	37.00 (24-62)	35.00 (26-56)	37.00 (25-57)	36.50 (24-62)		
High school graduate					0.149	0.928
No	92 (61.74)	18 (19.57)	21 (22.82)	53 (57.61)		
Yes	57 (38.26)	12 (21.05)	14 (24.56)	31 (54.39)		
Mean income from truck driving job per month (Kenyan Shillings)					2.565	0.277
8000–23,999 KES	34 (24.46)	4 (11.77)	9 (26.47)	21 (61.76)		
24,000–55,000 KES	105 (75.54)	26 (24.76)	24 (22.86)	55 (52.38)		
Married (legal or common-law)					NA	0.526**
No	25 (17.01)	5 (20.00)	8 (32.00)	12 (48.00)		
Yes	122 (82.99)	24 (19.67)	27 (22.13)	71 (58.20)		
Risk behavior						
Has other regular partner(s) on the trucking route					3.028	0.220
No	76 (51.70)	19 (25.00)	19(25.00)	38 (50.00)		
Yes	71 (48.30)	11 (15.49)	15 (21.13)	45 (63.38)		
Paid for sex in past 6 months					3.851	0.146
No	66 (47.14)	17 (25.76)	16 (24.24)	33 (50.00)		
Yes	74 (52.86)	10 (13.52)	17 (22.97)	47 (63.51)		
Always used condoms when had sex in the past 6 months					NA	0.292**
No	122 (84.72)	26 (21.31)	25 (20.49)	71 (58.20)		
Yes	22 (15.26)	4 (18.18)	8 (36.38)	10 (45.46)		
Drank alcohol in past year					6.296	0.043
No	72 (48.32)	18 (25.00)	21 (29.17)	33 (45.83)		
Yes	77 (51.68)	12 (15.59)	14 (18.18)	51 (66.23)		
Ever tested for HIV before					NA	0.278**
No	15 (10.07)	5 (33.33)	4 (26.67)	6 (40.00)		
Yes	134 (89.93)	25 (18.66)	31 (23.13)	78 (58.21)		
Number of years since last HIV test among those ever tested (those never tested assigned age)					2.161*	0.339*
Mean (SD)	4.74 (11.38)	6.07 (12.45)	5.41 (10.87)	3.98 (11.25)		
Median (range)	0.67 (0.1–59.0)	0.42 (0.08–42.92)	0.46 (0.08–39.00)	0.75 (0.08–59.00)		
Attitudinal scales						
Anticipated HIV stigma, higher score-more stigma (Cronbach’s alpha = 0.80)					4.308*	0.116*
Mean (SD)	0.80 (1.53)	0.97 (1.52)	1.03 (1.62)	0.64 (1.51)		
Median (range)	0.00 (0.0–9.00)	0.00 (0.00–6.00)	0.00 (0.00–6.00)	0.00 (0.00–9.00)		
Fatalism, higher score = more fatalistic (Cronbach’s alpha = 0.94)					5.745*	0.052*
Mean (SD)	46.62 (21.58)	44.17 (17.16)	54.57 (22.38)	44.18 (22.08)		
Median (range)	46.00 (20.00–97.00)	42.50 (20.00–93.00)	54.00 (20.00–97.00)	44.00 (20.00–92.00)		
General self-efficacy, higher score higher efficacy (Cronbach’s alpha = 0.90)					1.571*	0.456*
Mean (SD)	36.64 (4.40)	36.90 (3.22)	37.57 (3.84)	36.16 (4.92)		
Median (range)	39.00 (25.00–40.00)	37.00 (29.00–40.00)	39.00 (26.00–40.00)	39.00 (25.00–40.00)		
Gender-equity, higher score-more gender-equity attitudes (Cronbach’s alpha = 0.88)					4.841*	0.089*
Mean (SD)	58.36 (9.46)	60.60 (10.63)	56.20 (9.02)	58.46 (9.00)		
Median (range)	59.00 (34.00–72.00)	63.00 (34.00–72.00)	57.00 (35.00–72.00)	58.50 (38.00–72.00)		
Sensation-seeking, higher score = more sensation-seeking (Cronbach’s alpha = 0.73)					4.497*	0.106*
Mean (SD)	1.38 (0.60)	1.18 (0.33)	1.32 (0.52)	1.48 (0.69)		
Median (range)	1.00 (1.00–3.00)	1.00 (1.00–2.00)	1.00 (1.00–2.50)	1.00 (1.00–3.00)		

* Kruskal–Wallis Test

** Fishers exact test conducted in SAS 9.3 (Cary, NC)

Of the 149 participants, 20.13% refused HIV testing in the clinic, 23.49% chose the provider-administered blood test and 56.38% chose the self-administered oral test. There were no significant differences in test selected by any demographic variables. Among the HIV-related behaviors, only alcohol consumption in the past year was significantly associated with test selection. Specifically, those who had not consumed any alcohol in the past year were more likely to refuse testing (25.00 vs. 15.59% among those who consumed alcohol) or to select the provider-administered HIV test (29.17 vs. 18.18%) while those who reported drinking alcohol were more likely to choose the self-administered test (66.23 vs. 45.83%, p = 0.043). None of the attitudinal or belief scales were significantly associated with test selected, although the fatalism and gender-equity scales were of borderline significance. The mean score on the fatalism scale was higher among those who chose the provider-administered test compared with the self-administered test or no test (54.57, 44.18 and 44.17, respectively, p = 0.052); and the mean score on the gender-equity scale was lower among those who chose the provider-administered test compared to the self-administered test and no test (56.20, 58.45, and 60.60, respectively, p = 0.089). (Table 1).

### Regression Model Results Looking at Predictors of Choosing Supervised Self-administered Oral HIV Testing

In the crude models among those who tested in the clinic, which test was selected was significantly associated with alcohol consumption and fatalism scale score. Those who had consumed alcohol in the past year had odds of choosing the self-administered test over the provider-administered test 2.32 (95% CI: 1.04–5.19) times that of those who had not consumed alcohol in the past year; and for each additional unit on the fatalism scale score, the odds of choosing the self-administered test were 0.98 (95% CI: 0.96–0.99) times lower. Consistent condom use and higher general self-efficacy scale scores were both associated with lower odds of selecting the self-administered test but the associations were only of borderline significance (OR = 0.44, 95% CI: 0.16–1.24 and OR = 0.93, 95% CI: 0.84–1.02). In the multivariate model with all variables included, only scores on the fatalism, general self-efficacy scales and consistent condom use were significantly associated with selecting the self-administered test over the provider-administered test (OR = 0.96, 95% CI: 0.93–0.99; OR = 0.86, 95% CI: 0.73–1.03; OR = 0.16, 95% CI: 0.02–1.42). In the backwards stepwise regression model, only fatalism score, general self-efficacy score and consistent condom use remained in the final model (OR = 0.97, 0.94–0.99; OR = 0.83, 95% CI: 0.73–0.95; and OR = 0.34, 95% CI: 0.10–1.14, respectively). (Table 2).

**Table 2:** Logistic regression models looking at predictors of selecting the self-administered oral HIV test (versus the provider-administered blood test) among those who tested

	Crude models			Adjusted model (n = 98)		Likelihood ratio backward stepwise regression with p < 0.2 for remaining in the model) (n = 98)	
Variable	Number	OR (95% CI)	P-value	OR (95% CI)	P-value	OR (95% CI)	P-value
Age (years)	119	1.01 (0.96–1.07)	0.624	0.97 (0.90–1.1)	0.488		
High school graduate	119	0.88 (0.39–1.97)	0.751	0.74 (0.24–2.27)	0.601		
Income ≥24,000 KES/month from truck driving	109	0.98 (0.39–2.46)	0.969	1.23 (0.34–4.41)	0.748		
Married	118	1.75 (0.65–4.76)	0.270	0.49 (0.05–5.15)	0.551		
Have regular partners on trucking route in past 6 months (road wife)	117	1.50 (0.67–3.35)	0.322	1.66 (0.48–5.70)	0.421		
Paid for sex in past 6 months	113	1.34 (0.59–3.03)	0.481	0.78 (0.21–3.00)	0.731		
Always use condoms	114	0.44 (0.16–1.24)	0.120	0.16 (0.02–1.42)	0.100	0.34 (0.10–1.14)	0.081
Drank alcohol in past year	119	2.32 (1.04–5.19)	0.041	1.42 (0.49–4.14)	0.523		
Ever tested for HIV	119	1.68 (0.44–6.35)	0.447	NA	NA		
Years since tested for HIV	116	0.99 (0.96–1.02)	0.531	1.0 (0.98–1.07)	0.322		
Anticipated HIV stigma	119	0.86 (0.68–1.10)	0.224	0.80 (0.53–1.21)	0.289		
Fatalism	119	0.98 (0.96–0.99)	0.024	0.96 (0.93–0.0.99)	0.030	0.97 (0.94–0.99)	0.003
General self-efficacy	119	0.93 (0.84–1.02)	0.135	0.86 (0.73–1.03)	0.077	0.83 (0.73–0.95)	0.008
Gender-equity	119	1.03 (0.98–1.07)	0.214	0.98 (0.90–1.07)	0.692		
Sensation-seeking	119	1.52 (0.78–2.98)	0.219	1.38 (0.47–4.12)	0.560		

### Description of the Self-testing Procedures

Based on the observation checklist completed by the providers supervising the 84 participants who chose to self-administer the oral HIV test, more than half of participants (52.38%) completed the self-testing process without needing any guidance, while 47.61% asked questions during the self-testing and 13.10% needed the provider to intervene with correction because they were doing something incorrectly and did not ask for instruction. Steps where participants were more likely to need unsolicited correction included waiting for the full 20 min before reading the test result (6.17%) and interpreting the test result (5.00%). All of the tests were HIV-negative. At each step, about 20–30% of participants asked questions, with more asking questions about how to open the package and remove the materials (30.86%), locate and remove the testing swab without touching it (30.77%), waiting 20 min before viewing the test result (30.87%), and interpreting the test result (30.00%). (Table 3) Nearly all participants (97.40%) asked the provider to stay and view their test results with them, while a few (2.60%) viewed the results themselves but then disclosed the result during posttest counseling. (Data not shown).

**Table 3:** Steps in the self-testing process where participants needed guidance (asked questions or unsolicited correction from the provider) (n = 84)

	Did correctly without asking questions or needing correction	Did correctly but asked questions	Needed unsolicited correction by the provider to do correctly
Total^a^	44 (52.38%)	40 (47.62%)	11 (13.10%)
Looked at instructions provided	60 (73.17%)	22 (26.83%)	0 (0.00%)
Opened package and removed materials (3 missing)	54 (66.67%)	25 (30.86%)	2 (2.47%)
Removed cap on test tube (3 missing)	56 (69.14%)	24 (29.63%)	1 (1.23%)
Placed test tub in holder (3 missing)	58 (71.60%)	22 (27.16%)	1 (1.24%)
Located and removed the testing swab without touching it (missing 6)	52 (66.67%)	24 (30.77%)	2 (2.56%)
Collected oral sample (3 missing)	55 (67.90%)	23 (28.40%)	3 (3.70%)
Inserted swab into test tube (3 missing)	55 (67.90%)	24 (29.63%)	2 (2.47%)
Waited 20 min before reviewing for results (3 missing)	51 (62.96%)	25 (30.87%)	5 (6.17%)
Interpreted test correctly (4 missing)	52 (65.00%)	24 (30.00%)	4 (5.00%)

^a^All those who needed unsolicited correction also asked questions so the total is >84

### Regression Model Results Looking at Predictors of Needing Guidance When Self-administering the Oral HIV Test

In the crude models, those with higher anticipated stigma and higher fatalism scores were significantly more likely to need guidance when self-testing (OR = 1.64, 95% CI: 1.03–2.61; OR = 1.06, 95% CI: 1.03–1.08, respectively) while those with more gender-equitable scores had significantly lower odds of needing guidance (OR = 0.93, 95% CI: 0.88–0.99). In the adjusted model including all predictors, those with higher fatalism scores had higher odds of needing guidance (OR = 1.10, 95% CI: 1.03–1.17), while those with regular partners along the trucking route (road wives) had significantly lower odds of needing guidance (OR = 0.10, 95% CI: 0.01–1.00). In the final stepwise model, those with higher fatalism scores had significantly higher odds of needing guidance (OR = 1.07, 95% CI: 1.03–1.11). Those having regular partners along the trucking route (OR = 0.25, 95% CI: 0.06–1.00) had lower odds of needing guidance of borderline statistical significance, while being a high school graduate (OR = 2.93, 95% CI: 0.85–10.10) was associated with higher odds of needing guidance at borderline statistical significance. (Table 4).

**Table 4:** Logistic regression models looking at predictors of needing guidance (asking questions) when self-testing

	Crude models			Adjusted model (n = 69)		Likelihood ratio backward stepwise regression with p < 0.2 for remaining in the model) (n = 69)	
Variable	Number	OR (95% CI)	P-value	OR (95% CI)	P-value	OR (95% CI)	P-value
Age (years)	84	1.00 (0.95–1.05)	0.996	0.95 (0.85–1.05)	0.348		
High school graduate	84	1.58 (0.65–3.86)	0.312	2.05 (0.48–8.89)	0.336	2.93 (0.85–10.10)	0.089
Income ≥24,000 KES/month from truck driving	76	0.55 (0.20–1.54)	0.257	1.17 (0.26–5.38)	0.837		
Married	83	1.94 (0.53–7.04)	0.311	0.95 (0.05–18.01)	0.970)		
Have regular partners on trucking route in past 6 months (road wife)	83	0.89 (0.37–2.08)	0.762	0.10 (0.01–0.99)	0.049	0.25 (0.06–1.00)	0.050
Paid for sex in past 6 months	80	1.15 (0.47–2.81)	0.759	4.05 (0.40–31.19)	0.238		
Always use condoms	81	0.42 (0.10–1.74)	0.230	0.42 (0.02–8.83)	0.580		
Drank alcohol in past year	84	0.52 (0.21–1.25)	0.144	0.79 (0.19–3.34)	0.747		
Ever tested for HIV	84	0.16 (0.02–1.46)	0.105	NA	NA	NA	NA
Years since tested for HIV	82	1.04 (0.99–1.09)	0.167	1.03 (0.95–1.11)	0.541		
Anticipated HIV stigma	84	1.64 (1.03–2.61)	0.039	1.34 (0.72–2.49)	0.356		
Fatalism	84	1.06 (1.03–1.08)	<0.001	1.10 (1.03–1.17)	0.005	1.07 (1.03–1.11)	<0.001
General self-efficacy	84	0.92 (0.84–1.01)	0.085	1.14 (0.89–1.47)	0.341		
Gender - equity	84	0.93 (0.88–0.98)	0.011	1.07 (0.96–1.20)	0.237		
Sensation-seeking	84	1.72 (0.90–3.28)	0.102	1.76 (0.37–8.34)	0.476		

## Discussion

The majority of participants offered HIV testing choices chose the self-administered oral HIV test (56.38 vs. 23.49% who chose the provider-administered test and 20.13% who refused testing). Thus, the self-administered oral HIV test was acceptable to many. However, some participants still chose the standard blood-based provider-administered HIV test, suggesting that people differ in their testing preferences and choices are important so more people can find an option that works for them, as has been previously suggested [50].

When the truck drivers in this study self-administered the oral HIV test, the majority did so correctly without needing guidance (52.38%). However, a high proportion did ask questions during the process (47.61%) but by being allowed to ask questions, very few required unsolicited correction by the HTC (13.10%). In fact, all of those who needed unsolicited correction at some point in the testing process also asked questions at other points during the process, indicating that they knew they needed guidance to administer the test correctly, even if at some points when they needed guidance they did not request it while at other points they did. Thus it seems that, for the most part, people know when they do not understand the instructions and, if given an opportunity to ask questions to obtain clarification, most will be able to administer the test correctly. Therefore, it is important to have some mechanism for people to be able to ask questions while self-testing, especially that first time. This could be a phone hotline for populations who have access to phones, such as truck drivers, or offering supervised self-testing the first time someone tests him or herself.

The steps in the self-testing process where more participants asked questions or required unsolicited instruction included not contaminating the swab by touching it and waiting the necessary amount of time before looking at the test result. These issues have been reported in another study in Kenya where people were videotaped while self-administering the test [23], and therefore, the instructions for these steps need to be clarified or better emphasized. The issue of waiting time, in particular, needs better clarification, as some people may not have access to a dependable clock and, as found in our study, even those who do have such access may not monitor the time accurately. The fairly high proportion of participants who asked questions or required correction when interpreting the test results may be attributed in part to the fact that all the tests in this study were negative. Participants may have identified the negative result but leaned toward concluding that it was positive or inconclusive in the hope of receiving correction, which might be a stronger confirmation of a negative test than just having their reading of a negative test confirmed. This coping mechanism of expecting the worst even when it is unlikely has been described before [51].

Almost all of the participants who self-tested chose to view their test results with the HTC counselor. Of the two participants who opted to view their results alone, both disclosed the test result to the counselor during posttest counseling. This is important as one concern about self-testing is that people will not disclose their status and thus will not get appropriate counseling, referrals and linkage to HIV care. However, all participants who self-tested in this study tested HIV-negative and whether those who test HIV-positive who view their test results in private will also disclose that result during posttest counseling is unknown.

We found very few significant predictors of HIV test selection when offered HIV testing choices. None of the demographic variables differed among test refusers versus test accepters (either test), nor did they differ by the test selected (self- versus provider-administered) among those who did test. There were also few significant differences in testing or in the test selected among testers by HIV-related behaviors. Those who drank alcohol were significantly less likely to refuse HIV testing than to accept either testing choice, and among those who tested, drinkers were more likely to choose the self-administered test, but this association disappeared after adjusting for covariates. There were a few significant associations between test selected and psychosocial measures. Truck drivers have been described as having fatalistic views [7, 8], and it has been suggested that fatalism might be a barrier to HIV testing [52]. However, we found that fatalism scores were actually lowest among those who refused HIV testing compared to those who selected either the provider- or the self-administered HIV test, although the association was of borderline significance (p = 0.052). Among those who tested, higher fatalism scores were associated with significantly lower odds of choosing the self-administered oral HIV test over the provider-administered blood test in all regression models. This association was as we expected; as fatalism might impact concerns about severe reactions to the test result, we would expect that those with more fatalistic views would see more potential benefits for the provider-administered test rather than the self-administered test. The fact that the test refusers had lower fatalism scores than those who chose the self- or provider-administered test in the descriptive analysis might be due to the changing nature of the questions posed—from “would you like an HIV test?” to “which HIV test would you prefer?” When posed in terms of test preference rather than testing at all, those with more fatalistic views might have an easier time accepting one of the testing choices presented rather than actively refusing testing all together. It could also be that some of those who refused HIV testing did so because they wanted to test elsewhere, perhaps with a partner, which was a sentiment expressed by a number of study participants. Thus, some test refusers might be less fatalistic but have preferences for a different form of testing than what was offered in this study.

We were surprised to find that those with higher self-efficacy scores were less likely to choose the self-administered HIV test among those who tested. This association was only significant in the stepwise model, but was of borderline significance in the other models. We had expected that those with higher self-efficacy would be more likely to choose a test that they administer themselves rather than having someone else administer the test. A previous study in Malawi found self-efficacy to be positively associated with HIV testing in general [35], so again self-efficacy may have a different impact on test selection when given testing choices than when offered testing in a way that elicits a yes or no response. Also, it is important to note that our participants were recruited from a general healthcare clinic, although not necessarily there for HIV testing, and the impact of self-efficacy on HIV testing may be, in part, related to going to a clinic specifically for testing as opposed to accepting testing when offered after arrival at a clinic.

Although the sample was small (n = 69), we explored predictors of needing guidance while self-testing. The only significant association we found was that those with more fatalistic views had significantly higher odds of needing guidance and this association was consistent across all models (crude, multivariate and stepwise). This may be consistent with the idea that those who are more fatalistic have less faith that they have control over correctly administering the test and therefore seek guidance and affirmation that they are doing it correctly.

This study has a number of limitations. First, the number of participants included in these analyses was small (n = 149) and therefore we may not have had sufficient statistical power to identify some associations. The results presented here are for secondary research questions and the study was not powered specifically for these analyses. The lack of power was further exacerbated by the large number of variables we examined and when looking at a subset of the participants (e.g., the 69 who self-tested). We used backward stepwise regression as a way to try to reduce the number of variables in the model in the event that some were not significant in the full multivariate model due to over saturation. In addition, because of the number of variables examined, some associations found may have been spurious associations (i.e., type 1 errors). As all the independent variables were based on self-report, there may have been some misclassification due to recall error, social desirability bias or simply misunderstanding of the questions. The psychosocial scales administered have not been validated in Kenya and were new kinds of questions for our fieldworkers as well as for the participants and there may have been some discomfort or confusion in answering questions that were not obviously related to HIV, which may have impacted the quality of the responses. However, all of the scales demonstrated good internal consistency and a few were predictive of the outcomes examined, although not always in the direction expected. In addition, as study participants were recruited from clinics, our sample may not have been representative of truck drivers in Kenya or elsewhere, many of whom may not access clinics for healthcare services. Therefore, generalization of the results should be made with caution.

## Conclusions

Despite these limitations, our findings suggest that self-administered oral HIV testing was acceptable among this sample of Kenyan truck drivers and that when given the opportunity to ask questions, the vast majority (86.90%) were able to administer the test and interpret the results correctly themselves on the first try, and more than half of those who self-tested did so correctly without soliciting any guidance. Supervised self-testing, at least the first time someone uses the self-administered test, might be a good option to ensure correct use and provide the opportunity for asking questions. We did not find any differences in the HIV test chosen by demographic characteristics or HIV-related behavior. However, we did find an association between having more fatalistic views and lower odds of choosing the self-administered HIV test as well as higher odds of needing guidance when self-administering the test. This finding may warrant additional research into the impact fatalism plays on HIV testing behavior and what kinds of interventions can reduce fatalistic ideas around HIV and HIV testing, especially given the fact that truck drivers are viewed as being fairly fatalistic in their outlook [7, 8].
